# Berberine Acts on C/EBPβ/lncRNA Gas5/miR-18a-5p Loop to Decrease the Mitochondrial ROS Generation in HK-2 Cells

**DOI:** 10.3389/fendo.2021.675834

**Published:** 2021-08-30

**Authors:** Jiang Xu, Linqing Liu, Lin Gan, Yuanyuan Hu, Ping Xiang, Yan Xing, Jie Zhu, Shandong Ye

**Affiliations:** ^1^Department of Endocrinology, The First Affiliated Hospital of University of Science and Technology of China (USTC), Division of Life Sciences and Medicine, University of Science and Technology of China, Hefei, China; ^2^Department of Geriatrics, The First Affiliated Hospital of USTC, Division of Life Sciences and Medicine, University of Science and Technology of China, Hefei, China; ^3^Department of Microbiology, Anhui Medical University, Hefei, China; ^4^Department of Urology, The First Affiliated Hospital of USTC, Division of Life Sciences and Medicine, University of Science and Technology of China, Hefei, China

**Keywords:** diabetic nephropathy, berberine, reactive oxygen species, C/EBPβ, Gas5

## Abstract

**Background:**

Berberine (BBR) has therapeutic effect on diabetic nephropathy (DN), but its molecular mechanism is not completely clear.

**Methods:**

The DN model was established to observe the therapeutic effect of BBR. The expression levels of lncRNA Gas5 were detected by PCR. The transcriptional regulation of CCAAT enhancer binding protein beta (C/EBPβ) on Gas5 was analyzed by chromatin immunoprecipitation quantitative PCR (ChIP-qPCR) and luciferase reporter gene assay. The targeted regulation between Gas5 and miR-18a-5p and between miR-18a-5p and C/EBPβ 3′-untranslated region (3′-UTR) was also analyzed.

**Results:**

In HG environment, BBR decreased the mitochondrial reactive oxygen species (ROS) generation and activated the C/EBPβ expression in HK-2 cells; C/EBPβ could combine with the reaction element on the promoter of Gas5 to promote its expression. Gas5 also inhibited the miR-18a-5p expression as competing endogenous RNA (ceRNA) and reduce the negative regulatory effect of miR-18a-5p on C/EBPβ. BBR could activate C/EBPβ/peroxisome proliferator-activated receptor gamma coactivator 1-alpha (PGC-1α) signal pathway, regulate mitochondrial energy metabolism, and inhibit ROS production and apoptosis by activating C/EBPβ/Gas5/miR-18a-5p positive feedback loop in HG environment. It also showed that BBR alleviated streptozotocin (STZ) induced renal injury in DN rats *in vivo*.

**Conclusions:**

This study suggested that BBR could regulate the mitochondrial ROS generation by activating the positive feedback loop of C/EBPβ/Gas5/miR-18a-5p.

## Introduction

Diabetic nephropathy (DN) is an important chronic complication of diabetes. The formation of DN is related to dyslipidemia, hyperglycemia, hemodynamic changes, oxidative stress (OS), inflammatory response, and genetic susceptibility. The pathological changes include accumulation of extracellular matrix, thickening of glomerular basement membrane (GBM), glomerular hypertrophy, and glomerulosclerosis. The final manifestation is end-stage renal disease (1). The main clinical manifestations of DN are increasing urinary albumin excretion and decreasing glomerular filtration rate. Renal tubular injury has an important role in the progression of renal dysfunction (2). High glucose (HG) stimulation can induce physiological function and structural abnormalities of various renal tubular cells (RTC), including imbalance of renal tubular reabsorption, aging like lesions, apoptosis, and tubulointerstitial fibrosis of the epithelial cells, and even gradual progress to end-stage renal disease (1, 3). In diabetic HG environment, the expression of antioxidant enzymes in renal tubular endothelial cells was inhibited, and the generation of ROS and superoxide radicals in mitochondrial electron transport chain increased. Caspase family mediates apoptosis of proximal tubular epithelial cells, leading to tubular atrophy and renal failure. Therefore, the abnormal energy metabolism of mitochondria in RTC is an important reason for the occurrence and development of DN (4).

Berberine (BBR) is an isoquinoline alkaloid, which is the main active component of *Coptis chinensis* and Cortex phellodendron. *In vitro* and *in vivo* studies have shown that it has a good effect on reducing blood sugar, lipid, antioxidation, and inflammation (5). It was shown that BBR could reduce renal injury and inflammatory reaction in DN rats and reduce podocyte apoptosis induced by HG (6). BBR could also protect podocytes by inhibiting Drp1-mediated mitochondrion division and dysfunction (7). However, the molecular mechanism of the renal protective effect of BBR is still poorly understood.

Long noncoding RNAs (lncRNAs) are a kind of transcripts (larger than 200 bp); it does not encode proteins. lncRNAs have many biological functions, and their abnormal expression is related to many diseases, such as cancer, heart, nervous, and metabolic diseases (8). Some lncRNAs regulate gene expression by recruitment of transcriptional regulatory complexes with proximal (cis) or distal (trans) genomic binding sites (9); others can be used as scaffolds or decoys to assist in the binding of proteins or microRNAs (miRNAs) (10). A study has shown that a variety of lncRNAs are related to DN (11), but it is not clear whether BBR plays a therapeutic role through lncRNAs. Using a lncRNA expression chip to analyze the lncRNA expression level in the liver of nonalcoholic fatty liver disease (NAFLD) after BBR treatment, it was found that the 538 lncRNAs expression levels changed, among which MRAK052212 and MRAK080926, which are highly related to triglyceride metabolism, decreased after BBR treatment, while the decreased lncRNA MRAK052686 expression level increased after BBR treatment (12). We speculated that BBR may also play a role in renal protection by regulating the lncRNA expression in DN.

In this study, we selected 10 lncRNAs (13–20) related to the pathogenesis of DN and analyzed the expression levels of these 10 lncRNAs in HG and/or BBR treated HK-2 cells. It was found that the lncRNA Gas5 expression changed most obviously. BBR treatment or overexpression of Gas5 in HG-induced HK-2 cells could inhibit mitochondrial respiration and reduce ROS production. The results of this study suggested that BBR may regulate the expression of lncRNA to regulate the mitochondrial energy metabolism of RTC to alleviate renal injury in HG environment. At the same time, it enriches the transcriptional regulation spectrum of the key energy metabolism signal transduction pathway in renal tissue.

## Materials and Methods

### Samples

A total of 30 renal biopsy samples from DN patients and 30 surgical removal of normal tissue adjacent to renal cancer tissue from cancer patients were collected from January 2018 to August 2019 from the Nephrology and Urology Department of The First Affiliated Hospital of USTC (Hefei, China). Renal cancer patients with diabetes and hyperglycemia were excluded. Some tissues was frozen in a liquid nitrogen tank for subsequent nucleic acid extraction; the others were fixed and embedded, and paraffin section was prepared for H&E staining test. At the same time, the peripheral blood samples of the above 30 patients with DN and 30 healthy people from physical examination without diabetes and hyperglycemia history were collected, and the serum was used to detect the content of Gas5.

### Ethics Approval and Consent to Participate

All subjects signed the informed consent. These experiments were approved by the Ethics Committee of The First Affiliated Hospital of USTC.

### Cell Culture

HK-2 cells were obtained from the American Type Culture Collection (ATCC). They were cultured with Dulbecco’s modified Eagle’s medium (DMEM): F12 containing 15 mM N-2-hydroxyethylpiperazine-N-2-ethanesulfonic acid (HEPES), L-glutamine, and pyridoxine HCl, supplemented with 10% (v/v) fetal calf serum (FCS) at 37°C with 5% CO_2_ and 95% humidified air. An HG injury cell model was established with 56 mM glucose; the cells were treated with 10 μM BBR. The cells were divided into HG group (HG), control group (NG), BBR-treated normal cell group (NG + BBR), and BBR-treated HG cell group (HG + BBR) randomly.

### Cell Transfection

Lentivirus expressing CCAAT enhancer binding protein beta (C/EBPβ), lncRNA Gas5, miR-18a-5p, and their corresponding short hairpin RNA (shRNA) was obtained from Hanheng Biotechnology Co., Ltd. They were transfected into HK-2 cells, respectively, by Lipofectamine 3000 according to the manual. Stable cell lines with stable or downregulated genes were selected by adding puromycin (Sigma-Aldrich, MO, USA).

### Experimental Animals

Forty SD rats (Anhui Experimental Animal Center, Hefei, China) were divided into four groups randomly. Twenty of them were fed with high-fat diet (48% carbohydrate, 22% fat, and 20% protein with total calorific value of 44.3 kJ/kg) for 4 weeks. Streptozotocin (STZ) was injected at the dose of 60 mg/kg intraperitoneally on an empty stomach. The normal rats were injected with the same amount of 0.1 mmol/L sterile citrate buffer. The blood glucose was tested after 5 days. The diabetes model was considered to be established successfully if the blood glucose was more than 16.7 mmol/L. Diabetic rats were still fed with high-fat diet for 1 week. They were divided into model group (DN group) and DN + BBR group [BBR dissolved in 0.5% carboxymethyl cellulose, gavage at the rate of 100mg/kg day (21) for 8 weeks], with 10 rats in each group. The normal feeding rats were divided into control group (NC group), NC + BBR group (BBR dissolved in 0.5% carboxymethyl cellulose, gavage with 100 mg/kg day for 8 weeks), 10 rats in each group. At the eighth week, the blood glucose levels were detected. Urine samples were collected to detect creatinine and urinary albumin, and then, the ratio of urinary albumin to urinary creatinine was calculated. Then, the rats in each group were anesthetized, and the renal tissues were fixed with 4% neutral formaldehyde for pathological and immunohistochemical staining. Two rats died in DN group at the end of 8 weeks; it may be that they were intolerant to the blood glucose level that changes too fast in a short time.

### Renal Function Test

Urinary albumin was measured with rat urinary albumin radioimmunoassay kit according to the kit’s instructions. Urine creatinine was detected by the kit according to the manual. The urinary albumin-to-creatinine ratio (UACR) was calculated to eliminate the effect of urine dilution: UACR (mg/g) = Ualb (mg/L)/[Ucr (μmol/L) × the Cr molecular weight (113.12 g/mol) × 10^−6^].

### HE, PAS Staining, and Immunohistochemical Detection of Renal Tissue

The renal samples were fixed with 4% paraformaldehyde; they were embedded in paraffin and cut into 4 μm tissue sections. HE and periodic acid–Schiff (PAS) staining were performed with HE and PAS staining kits (Solarbio, Beijing, China) according to the instructions, respectively. PAS-stained sections were observed and photographed with a light microscope by the pathologists. The percentages of PAS positive area and glomerular area were analyzed by ImageJ software to quantitatively analyze the expansion of glomerular mesangial matrix (22).

The expression of C/EBPβ in kidney tissues of different groups was detected by immunohistochemistry. After the paraffin sections were dewaxed and dehydrated in xylene and gradient ethanol, high-pressure antigen repair was performed with 0.01M sodium citrate buffer solution for 15 min. They were incubated with 3% hydrogen peroxide (H_2_O_2_) for 10 min; then, they were washed with 0.02M phosphate-buffered saline (PBS) (pH 7.2) for three times and 3 min each time. The anti-C/EBP β antibody (Abcam, ab264305, 1:500) was added and incubated in a wet box for 2 h at room temperature. They were washed with PBS for three times, and goat antirabbit immunoglobulin G–horseradish peroxidase (IgG-HRP) (Abcam, ab205718, 1:5,000) was added into them. They were incubated for 1 h at room temperature and washed with PBS for three times; 3,3′-diaminobenzidine (DAB) solution was added and incubated in the dark for 5–15 min. They were stained with hematoxylin for 3 min and dehydrated with gradient alcohol and permeabilized with xylene after washing; the sections were sealed with neutral resin and observed under a microscope (Nikon, Japan) and photographed.

### RNA Fluorescence *In Situ* Hybridization

We extracted DNA from HK-2 cells as PCR template. Sense or antisense RNA probes were labelled by Digoxigenin, they were synthesized by MAXIscript T3 and T7 kit using the T3 and T7 promoters in the blunt vector. Fluorescence *in situ* hybridization (FISH) assay was performed according to the published protocol (22). Briefly, the cells in different groups were fixed with 4% paraformaldehyde. They were permeabilized in the solution (1:1 acetone/methanol), and then, they were hybridized with Gas5 sense or antisense RNA probes labeled by digoxigenin. The hybridized sections were incubated with antidigoxigenin antibody conjugated with peroxidase after blocking. They were revealed by SuperGloTM Green Immunofluorescence Amplification Kits. Nuclei were counterstained using 4′,6-diamidino-2-phenylindole (DAPI) (Beyotime Biotechnology, Shanghai, China). Images were collected by a Nikon80i immunofluorescence microscope (Nikon, Japan).

### RNA Extraction and qRT-PCR

Total RNA of serum was extracted with Serum RNA Purification Midi Kit following the instructions. The detection of Gas5 in serum was performed according to (17). Cells’ RNA was extracted by the Trizol reagent kit. RNA (1 μg) was subjected to reverse transcription by MMLV Reverse Transcriptase Kit (Takara Bio Co., Ltd., Dalian, China). The expression of miR-18a-5p messenger RNA (mRNA) was detected by GenePharma Hairpin-it Kit according to the manufacturer’s instructions. lncRNAs and C/EBPβ mRNA expression levels were determined by SYBR Premix Kit according to the kit’s instructions. The parameters were 95°C for 10 min and 40 cycles of 95°C for 10 s, 60°C for 20 s, and 72°C for 20 s. A melting curve analysis was conducted at the end of each reaction. Normalization of RNA was performed using U6 and glyceraldehyde 3-phosphate dehydrogenase (GAPDH) as internal control. Quantifications was performed by the 2^−ΔΔCt^ method. Primers’ sequences are listed in **Table 1**.

**Table 1:** Primers’ sequences used in this study.

Gene	Forward primer(5′–3′)	Reverse primer(5′–3′)
Blnc1	TCTCCAACCATCTGCCTTAT	TCCCTCTGCCTCTGACCT
PRINS	CAGCAGACAGGAGCAAGG	AAGAAGCGACTGGGAACA
LINK-A	GCCATCAAACTCCAACCA	AAGCGTAAGAATGAAGACCA
Tug1	GACCCAGAAGAGTTAAGAATC	CAGAATAGAAGCCAAGCAG
Gas5	AAAGAGCAAGCCTAACTCA	TTACCAGGAGCAGAACCA
MALAT1	ACCTAACCAGGCATAACA	AGTAGACCAACTAAGCGAAT
H19	CTGGGCAACGGAGGTGTA	TCTGCTGGGAGGGTGTCTG
ARAP1-AS2	TTGCCACTACTGATTCACTCT	CACATCCGTTTCTCACCTC
ARAP1-AS1	GAAGGAAGGCTGAAGTCCCT	GAAGGAAGGCTGAAGTCCCT
PVT1	TCAGCACTCTGGACGGACTT	ATGGCATGGGCAGGGTAG
PGC-1α	TCTGAGTCTGTATGGAGTGACAT	CCAAGTCGTTCACATCTAGTTCA
C/EBPβ	CTTCAGCCCGTACCTGGAG	GGAGAGGAAGTCGTGGTGC
GAPDH	GCTCTCTGCTCCTCCTGTTC	ACGACCAAATCCGTTGACTC
miR-18a-5p	CGCGTAAGGTGCATCTAGTGC	AGTGCAGGGTCCGAGGTATT
miR-185-5p	CGCGTGGAGAGAAAGGCAGT	AGTGCAGGGTCCGAGGTATT
miR-188-5p	GCGCATCCCTTGCATGGT	AGTGCAGGGTCCGAGGTATT
miR-345-3p	GGCCCTGAACGAGGGGT	AGTGCAGGGTCCGAGGTATT
miR-501-3p	GAATGCACCCGGGCAAG	AGTGCAGGGTCCGAGGTATT
U6	CTCGCTTCGGCAGCACA	AACGCTTCACGAATTTGCGT

### Mitochondrial ATP Determination

Cellular ATP levels were measured using the Cell Viability Assay Kit as previously described (23).

### Mitochondrial ROS Determination

HK-2 cells were serum starved overnight when they were 70% confluent in six-well plates followed by treatment with NG, HG, NG + BBR, or HG + BBR for 48 h. The cells were cultured at 37°C with fresh media containing 5 μM MitoSox Red mitochondrial superoxide indicator (Invitrogen) for 10 min. The mean fluorescence intensity (MFI) was detected by flow cytometry.

### Cell Apoptosis Detection

The apoptosis was detected using Annexin V-PI Analysis Kit following the manual. HK-2 cells were serum starved overnight when they were 70% confluent in six-well plates followed by treatment with NG, HG, NG + BBR, or HG + BBR for 48 h. The cells were digested and harvested. They were washed with precooled PBS thrice, binding solution (195 μl Annexin V-FITC) was added, and the cells were resuspended gently. Annexin V-FITC (5 μl) was added and mixed gently. The staining solution (10 μl PI) was added. The cells were incubated at room temperature (RT) (20–25°C) in the dark for 20 min; they were placed in ice bath and detected by a flow cytometer.

### Western Blotting Test

The total proteins were extracted from different groups; bicinchoninic acid (BCA) kit was used to determine protein concentration. Proteins were separated by 12% sodium dodecyl sulfate–polyacrylamide gel electrophoresis (SDS-PAGE). They were electrotransferred to a polyvinylidene fluoride (PVDF) membrane and rinsed for 15 min with Tris-buffered saline (TBS). It was blocked, and appropriate dilution of primary antibodies [C/EBPβ, CST, #90081, 1:1,000; peroxisome proliferator-activated receptor gamma coactivator 1-alpha (PGC-1α), Novus, NBP1-04676, 1:1,000] were added and incubated at 4°C overnight. The membrane was rinsed and then incubated with secondary antibody at RT for 1 h. The bands were determined by enhanced chemiluminescence kit. Imagequant LAS4000 (GE Healthcare, Japan) was used to observe them.

### Chromatin Immunoprecipitation-qPCR Assay

Chromatin isolation by RNA purification (ChIRP) was performed with SimpleChIP^®^ Kit according to the kit’s protocol. Briefly, the chromatin was segmented by ultrasonic treatment after 1 × 10^7^ cells were cross-linked by formaldehyde, then incubated with antihuman C/EBPβ antibody (CST, #90081) and antihuman PGC-1α antibody (Novus, NBP1-04676) at 4°C overnight. Histone H3 (D2B12) XP^®^ Rabbit mAb (CST, #4620) was used as the positive control, and normal rabbit IgG (CST, #2729) was the negative control. ChIP-Grade Protein G magnetic beads were added into them, and they were incubated at 4°C for 2 h; DNA on the beads was eluted. The standard curve was generated by real-time fluorescent quantitative PCR with continuous diluents (undiluted, 1:5, 1:25, 1:125) of 2% input chromatin DNA, and the enrichment degree of Gas5 promoter DNA in different groups of samples was determined.

### Double Luciferase Reporter Gene Analysis

To analyze the regulatory effect of PGC-1α on Gas5 promoter, we inserted 2,000 nt upstream and 100 nt downstream of Gas5 transcription start site into pGL3-Basic plasmid (Promega, e1751) to construct pGL3-Basic-Gas5 plasmid. pex-3-PGC-1α (gene Pharma) plasmid expressing PGC-1α was cotransfected into HEK293 cells with pGL3-Basic-Gas5 and pGL3-Basic, respectively. The cells were split by Dual Luciferase Reporter System following the protocol after culture for 48 h. Luminescence was added and was detected by a Panomics luminometer.

To analyze the regulatory effect of C/EBPβ on Gas5 promoter, we constructed wild-type pGL3-Basic-Gas5 (ATTGC) plasmid and mutant pGL3-Basic-Gas5 (AAAAA) plasmid based on the sequence of responsive element on Gas5 promoter. pex-3-C/EBPβ (gene Pharma) plasmid expressing C/EBPβ was cotransfected into HEK293 cells with pGL3-Basic-Gas5 (ATTGC), pGL3-Basic-Gas5 (AAAAA), and pGL3-Basic, respectively. The cells were split by Dual Luciferase Reporter System following the protocol after culture for 48 h. Luminescence was added and was detected by the Panomics luminometer.

To analyze the targeting effect of Gas5 and miR-18-5p, we constructed wt-pGL3-Gas5 and mut-pGL3-Gas5 luciferase reporter genes based on the prediction of the binding site of Gas5 and miR-18-5p; they were cotransfected into HEK293 cells with miR-18-5p and Renilla luciferase. The cells were split by Dual Luciferase Reporter System following the protocol after culture for 48 h. Luminescence was added and was detected by the Panomics luminometer. The internal reference was sea renin fluorescence.

To analyze the interaction of miR-18-5p and the 3′-untranslated region (3′-UTR) of C/EBPβ, we constructed mutant and wild-type C/EBPβ 3′-UTR luciferase reporter gene plasmids mut-pGL3-C/EBPβ and wt-pGL3-C/EBPβ. Luciferase reporter plasmid, miR-18-5p mimic or control, and Renilla luciferase were transfected into 293 cells simultaneously. The cells were split by Dual Luciferase Reporter System following the protocol after culture for 48 h. Luminescence was added and was detected by the Panomics luminometer. The sea renin fluorescence was the internal reference.

### RNA Immunoprecipitation *A*ssay

RNA immunoprecipitation (RIP) assay was conducted using the EZMagna RIP kit. The cells were harvested when they grew to 80%–90% full. They were lysed by RIP lysis buffer. They were coincubated with magnetic beads combined with anti-Ago2 antibody, 100 μl cell lysate, and negative control normal mouse IgG or anti-SNRNP70. To remove the protein, the proteinase K was added, and they were incubated at 55°C for 30 min. RNA was purified using RNeasy Micro Kit. The purity and content of RNA was detected with a NanoDrop™ 1000 spectrophotometer (Thermo Fisher Scientific). The coprecipitated RNAs were detected by RT-PCR.

### Statistical Analysis

SPSS 20.0 software was used to analyze the data. The differences were evaluated by Student’s t-test or one-way ANOVA. The correlation between two genes was tested by Spearman’s correlation test. p < 0.05 was significant.

## Results

### BBR Regulated HK-2 Cells Mitochondrial ROS Generation Under HG Environment

**Figure 1:**
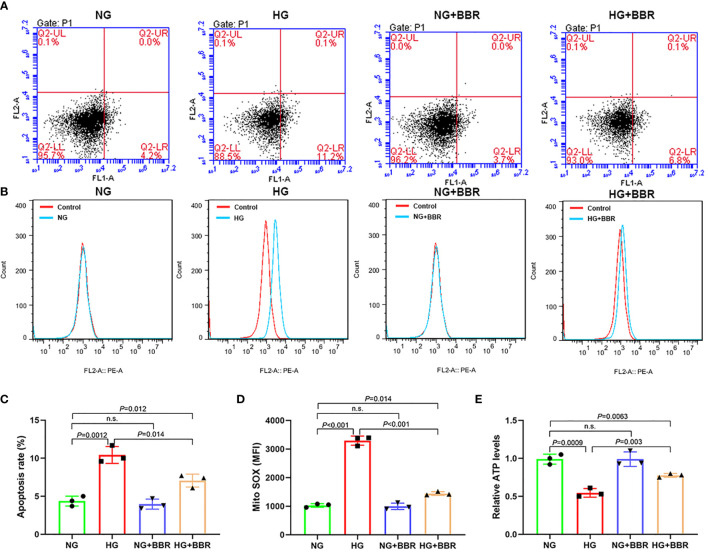
BBR could regulate the mitochondrial bioenergetics of HK-2 cells under HG condition. **(A, C)** The effect of BBR on the apoptosis of HK-2 cells under HG condition detected by flow cytometry. **(B, D)** The effect of BBR on the mitochondrial ROS under HG condition detected by flow cytometry. **(E)** Relative ATP content in HK-2 cells of different groups. Cell culture experiments were repeated at least three times. n.s., no significance.

We observed the effect of BBR on the mitochondrial ROS generation of HK-2 cells in HG environment. Under HG condition, the apoptosis level (**Figures 1A, C**) and mitochondrial ROS level (**Figures 1B, D**) of HK-2 cells were significantly increased, while BBR could downregulate the apoptosis and mitochondrial ROS level. ATP level of HK-2 cells decreased under HG condition, while BBR could upregulate ATP level (**Figure 1E**). BBR had no significant effect on mitochondrial ROS level in HK-2 cells in normal glucose environment (NG). These suggested that BBR could regulate the mitochondrial ROS level of HK-2 cells in HG condition.

### BBR Upregulated the lncRNA Gas5 Expression in HG-Cultured HK-2 Cells

**Figure 2:**
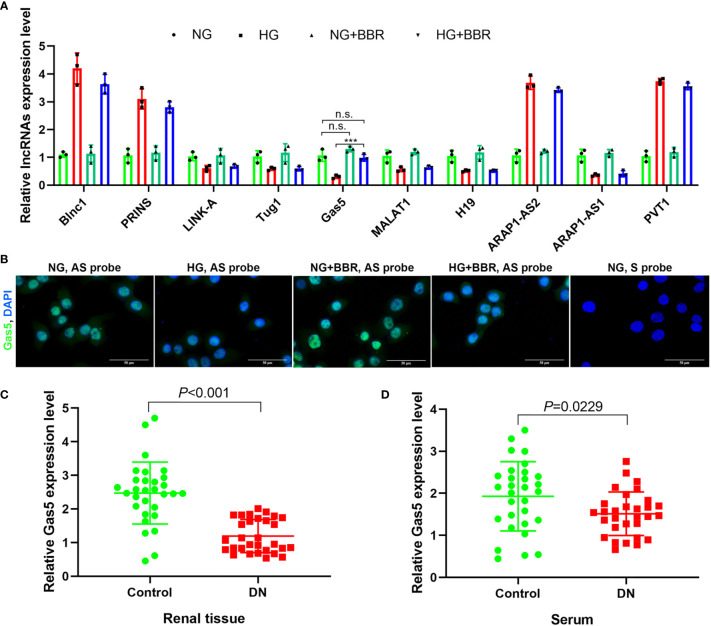
lncRNA Gas5 may be the therapeutic target of BBR. **(A)** The expression level of 10 lncRNAs in different groups was analyzed by RT-PCR. **(B)** RNA FISH results of the HG effect on the Gas5 expression in HK-2 cells. **(C)** The Gas5 expression was detected in renal biopsy specimens of DN patients and paracancerous tissues of renal cancer patients by RT-PCR. **(D)** Gas5 expression in serum of DN patients and healthy people. ***p < 0.001. n.s., no significance.

The expression levels of reported 10 DN-related lncRNAs treated by HG and BBR were analyzed by RT-PCR. It was found that the Gas5 expression level was most regulated by BBR (**Figure 2A**) under HG condition. RNA FISH results also showed that Gas5 was distributed in the nucleus and cytoplasm of HK-2 cells; the expression level of Gas5 in HG decreased significantly, while BBR could upregulate its expression (**Figure 2B**). The Gas5 expression in renal tissue (**Figure 2C**) and serum (**Figure 2D**) of DN patients was significantly decreased compared with the control group. These results indicated that the expression level of lncRNA Gas5 in HK-2 cells and renal tissue decreased in HG environment. BBR could upregulate the Gas5 expression in HG-cultured HK-2 cells. Gas5 may be the therapeutic target of BBR.

### BBR Regulated Mitochondrial ROS Level of HK-2 Cells in HG Environment Dependent on Gas5

**Figure 3:**
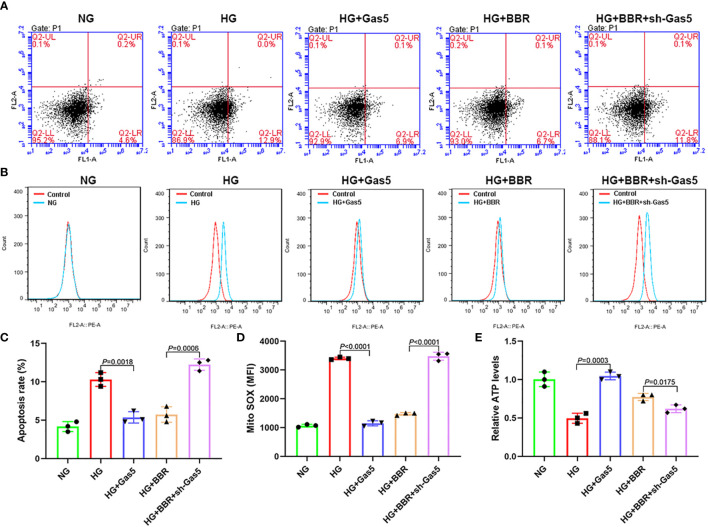
BBR regulated mitochondrial ROS level of HK-2 cells in high glucose environment dependent on Gas5. **(A, C)** The proportion of apoptosis in different groups detected by flow cytometry. **(B, D)** Mitochondrial ROS in different groups detected by flow cytometry. **(E)** Relative ATP content in HK-2 cells of different groups.

In order to observe the Gas5’s role in mitochondrial ROS generation of HK-2 cells, we overexpressed Gas5 in the HG group and downregulated Gas5 expression in HG + BBR group cells. The results showed that in the HG + Gas5 group, the ratio of apoptosis and the mitochondrial ROS level were significantly decreased, and ATP level were significantly increased when compared with HG group (**Figure 3**). However, compared with the HG + BBR group, in the HG + BBR + sh-Gas5 group, the apoptosis ratio and mitochondrial ROS level were significantly increased, and ATP level was significantly reduced (**Figure 3**). Compared with the HG + BBR group, the apoptosis and ROS level decreased in the HG + BBR + Gas5 group, but there was no statistical difference. These suggested that BBR regulated mitochondrial ROS level by promoting the expression of Gas5.

### BBR Relied on C/EBPβ to Promote the Gas5 Expression

**Figure 4:**
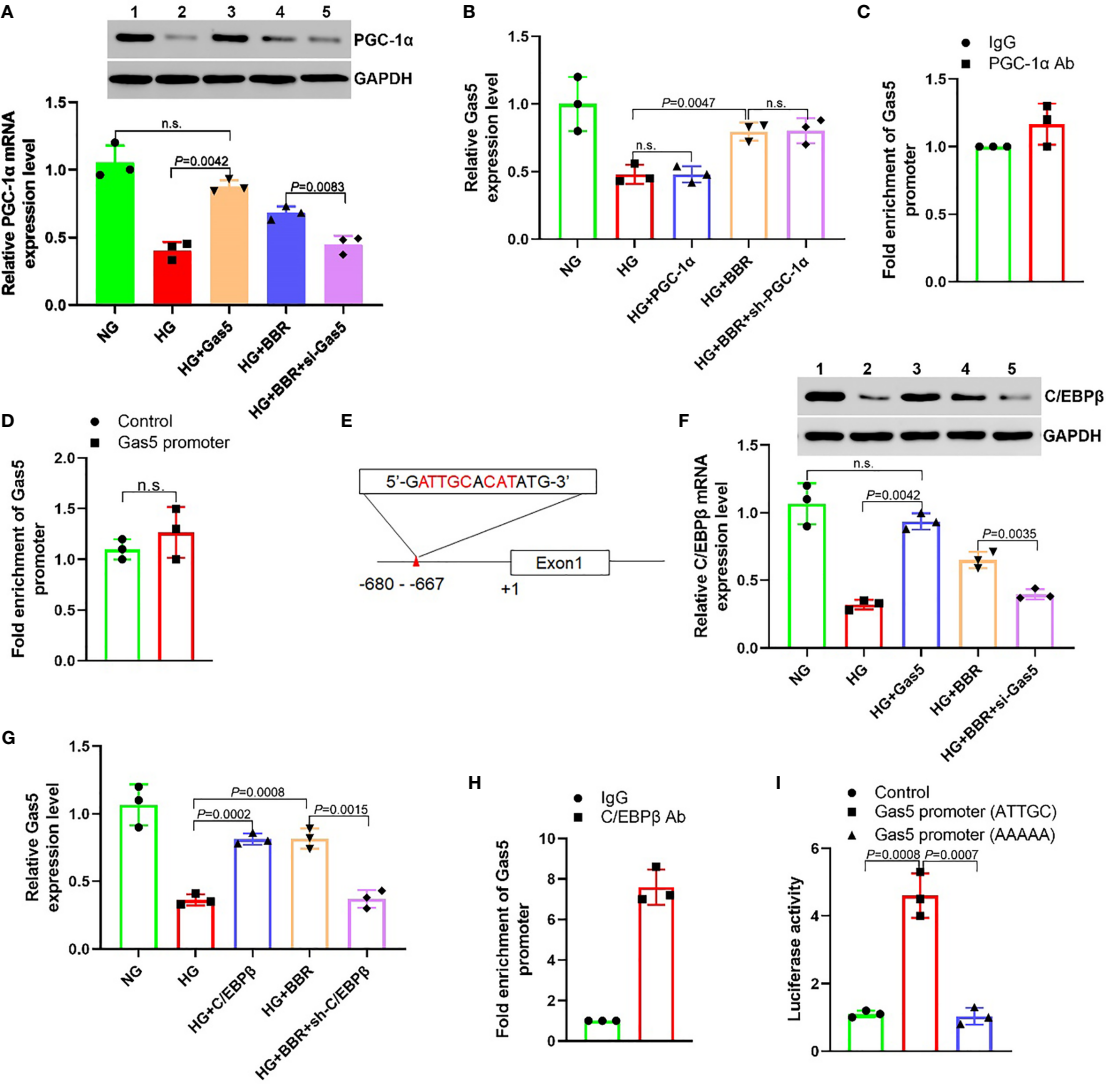
BBR regulates the expression of Gas5 dependent on C/EBPβ. **(A)** The effect of BBR and Gas5 on the PGC-1α expression. **(B)** The effect of PGC-1α on the Gas5 expression. **(C)** Analysis of the effect of PGC-1α on Gas5 promoter by ChIP-qPCR. **(D)** The effect of PGC-1α on Gas5 promoter. **(E)** Schematic illustration of consensus C/EBPβ responsive element in Gas5 gene promoter. **(F)** The effect of BBR and Gas5 on the C/EBPβ expression. **(G)** The effect of C/EBPβ on the Gas5 expression. **(H)** Analysis of the effect of C/EBPβ on Gas5 promoter by ChIP-qPCR. **(I)** The effect of C/EBPβ on Gas5 promoter. n.s., no significance.

Both BBR and Gas5 are related to the mitochondrial ROS generation of HK-2 cells. PGC-1α is one of the key molecules involved in the regulation of mitochondrial energy metabolism (24). This study found that HG could inhibit the PGC-1α expression. Both BBR and overexpression of Gas5 could upregulate the PGC-1α expression (**Figure 4A**). However, up- or downregulation of PGC-1α expression in HK-2 cells had no significant effect on the Gas5 expression (**Figure 4B**). ChIP-qPCR results showed that PGC-1α antibody could not enrich the DNA of the Gas5 promoter region (**Figure 4C**). The results of the luciferase reporter gene also showed that PGC-1α could not regulate Gas5’s promoter activity (**Figure 4D**). We predicted the transcription factors binding to the Gas5 promoter (alggen.lsi.upc.es/cgi-bin/promo_v3/promo/promoinit.cgi?dirDB=TF_8.3); it was found that C/EBPβ had a higher score (**Figure 4E**). A study showed that C/EBPβ could directly activate the PGC-1α expression (25). This study showed that HG inhibited the C/EBPβ expression, while BBR and overexpression of Gas5 could upregulate the C/EBPβ expression (**Figure 4F**). Upregulation of C/EBPβ could promote the Gas5 expression; downregulation of C/EBPβ could inhibit the expression of Gas5 (**Figure 4G**). ChIP results showed that C/EBPβ antibody could enrich the DNA of Gas5 promoter region (**Figure 4H**). It was found that C/EBPβ could activate the promoter activity of wild-type (ATTGC) Gas5. However, it had no effect on the mutant (AAAAA) Gas5 promoter activity (**Figure 4I**). These results showed that BBR regulated the Gas5 expression relying on C/EBPβ; C/EBPβ could directly promote the Gas5 expression as a transcription factor. At the same time, it was also observed that Gas5 could promote the expression of C/EBPβ and PGC-1α. Since PGC-1α could not directly regulate the Gas5 expression, we speculated that there may be feedback regulation between C/EBPβ and Gas5.

### BBR Regulated the Mitochondrial ROS Levels of HK-2 Cells in HG Environment Dependent on C/EBPβ

**Figure 5:**
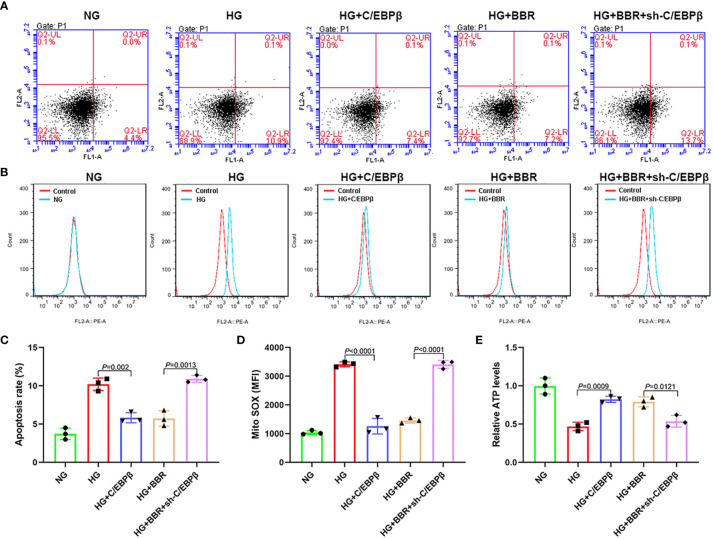
BBR relies on C/EBPβ to regulate mitochondrial ROS levels of HK-2 cells in high glucose environment. **(A, C)** The apoptosis ratio in different groups. **(B, D)** Mitochondrial ROS in different groups. **(E)** Relative ATP content in HK-2 cells of different groups.

To observe the role of C/EBPβ in mitochondrial ROS generation of HK-2 cells, we overexpressed C/EBPβ in the HG group cells and downregulated C/EBPβ in the HG + BBR group cells. The results showed that in the HG + C/EBPβ group, the apoptosis ratio and ROS level of mitochondria decreased significantly, and the ATP level increased significantly compared with that of HG group (**Figure 5**). Compared with that of the HG + BBR group, the apoptosis ratio and mitochondrial ROS level were increased significantly, and the ATP levels were reduced significantly in the HG + BBR + sh-C/EBPβ group (**Figure 5**). These suggested that BBR played a role in mitochondrial ROS level regulation by promoting the C/EBPβ expression.

### Gas5 as ceRNA Regulated the miR-18a-5p and C/EBPβ Expression

**Figure 6:**
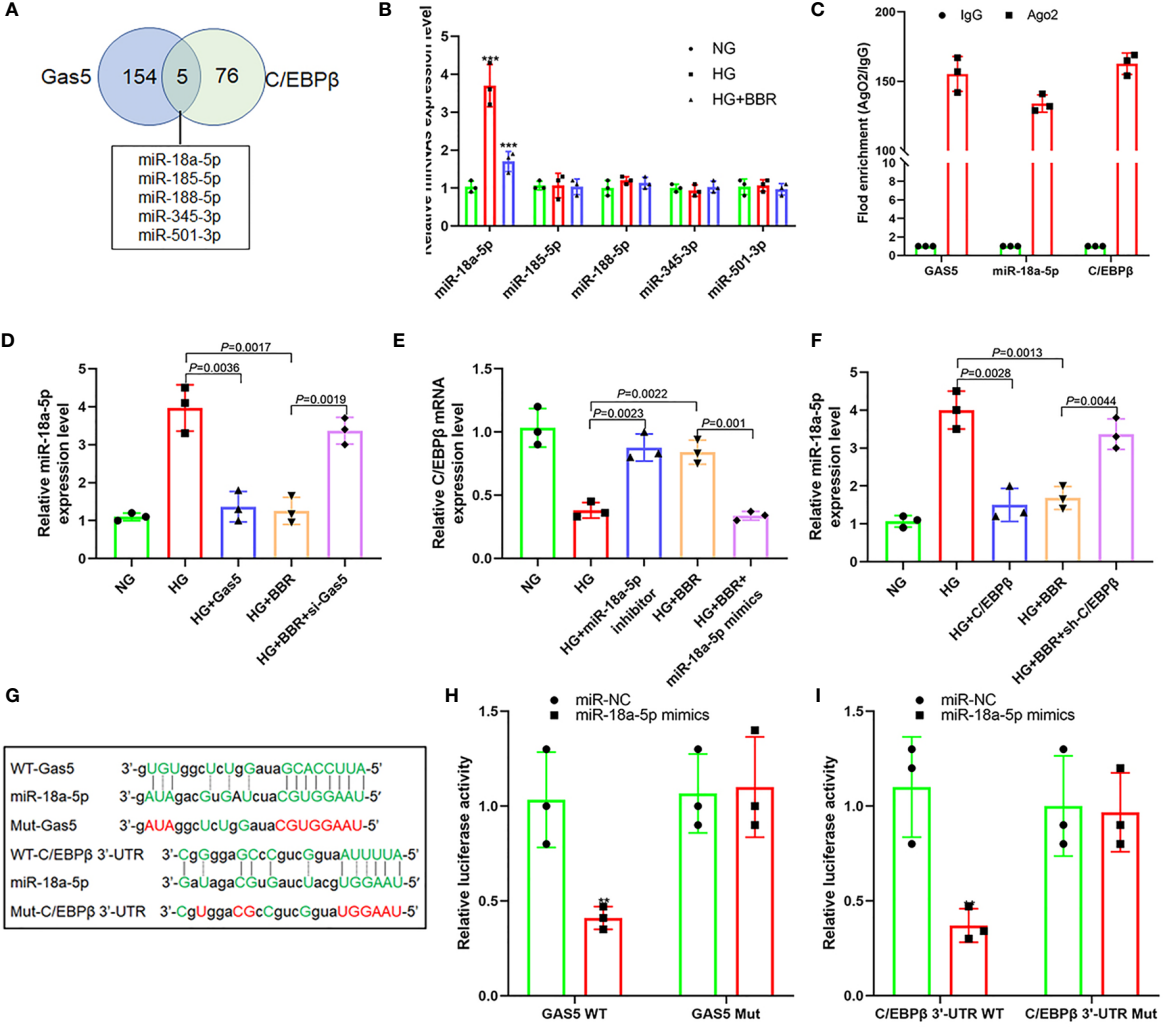
Gas5 regulated the expression of miR-18a-5p and C/EBPβ as ceRNA. **(A)** Five miRNAs that can simultaneously bind with Gas5 and C/EBPβ 3′-UTR, and the intersection is shown as a Venn plot. **(B)** Effect of BBR on the expression of five miRNAs in HK-2 cells under HG condition (***P < 0.001). **(C)** RIP assay was used for detection of the enrichment of miR-18a-5p, Gas5, and C/EBPβ in response to anti-Ago2 compared to the negative control IgG. **(D)** Effect of HG and Gas5 on the expression of miR-18a-5p. **(E)** Effect of miR-18a-5p on the C/EBPβ mRNA expression. **(F)** Effect of C/EBPβ on the miR-18a-5p expression. **(G)** Binding sites of miR-18a-5p, Gas5, and C/EBPβ 3′-UTR and construction of plasmids. **(H)** Targeting effect of miR-18a-5p and Gas5 (**P < 0.01). **(I)** Targeting effect of miR-18a-5p and C/EBPβ 3′-UTR (**P < 0.01).

It was found that C/EBPβ upregulated the Gas5 expression, and Gas5 could also upregulate the C/EBPβ expression; there may be feedback regulation between C/EBPβ and Gas5 (**Figures 4F, G**). lncRNA could be used as ceRNA to absorb and inhibit target gene expression miRNA to upregulate gene expression indirectly. Thus, we predicted all miRNAs capable of binding to the 3′-UTR of Gas5 and C/EBPβ using ENCORI (starbase.sysu.edu.cn) and miRWalk 3 (http://mirwalk.umm.uni-heidelberg.de/). It was found that five miRNAs maybe combine with C/EBPβ and Gas5 simultaneously (**Figure 6A**). qPCR results showed that the miR-18a-5p expression in HG was significantly higher than that in NG, while the miR-18a-5p expression decreased significantly in the HG + BBR group; the other four miRNAs did not change significantly (**Figure 6B**). RIP results found that miR-18a-5p, Gas5, and C/EBPβ mRNA were enriched in the precipitate (**Figure 6C**), which indicated that there was targeted binding among them. The miR-18a-5p expression increased in the HG group cells, and overexpression of Gas5 could inhibit its expression. In the HG + BBR group, the miR-18a-5p expression decreased; interference with Gas5 could promote its expression (**Figure 6D**). miR-18a-5p inhibitor transfection in the HG group could upregulate the C/EBPβ mRNA expression, while miR-18a-5p mimics transfection in the HG + BBR group could inhibit the C/EBPβ mRNA expression (**Figure 6E**). Overexpression of C/EBPβ in the HG group could inhibit the miR-18a-5p expression, and downregulation of C/EBPβ in the HG + BBR group could upregulate the miR-18a-5p expression (**Figure 6F**). Therefore, an interaction existed between C/EBPβ, Gas5, and miR-18a-5p.

Further analysis of their gene sequences found that targeted regulatory sites existed between the 3′-UTR of Gas5 and miR-18a-5p and between the 3′-UTR of miR-18a-5p and C/EBPβ (**Figure 6G**). **Figure 6H** also shows that there was targeted regulation between the 3′-UTR of Gas5 and miR-18a-5p and between the 3′-UTR of miR-18a-5p and C/EBPβ (**Figure 6I**). These indicated that BBR may promote the upregulation of Gas5 expression level by activating C/EBPβ in HK-2 cells under the HG condition. As a ceRNA, Gas5 inhibited the expression level of miR-18a-5p, which further promoted the C/EBPβ expression and formed a loop.

### The Regulation of BBR on Mitochondrial ROS Levels of HK-2 Cells in HG Environment Was Affected by the miR-18a-5p Expression Level

**Figure 7:**
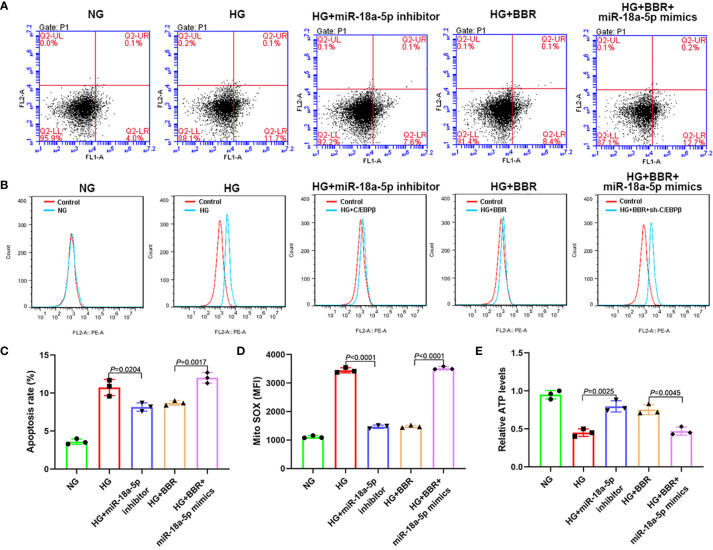
The expression level of miR-18a-5p was related to the mitochondrial ROS levels of HK-2 cells in high glucose environment. **(A, C)** The apoptosis ratio in different groups. **(B, D)** Mitochondrial ROS levels in different groups. **(E)** Relative ATP content in HK-2 cells of different groups.

As shown in **Figure 7**, in the HG + miR-18a-5p inhibitor group, the proportion of apoptosis and the mitochondrial ROS level were significantly decreased; the ATP levels were increased significantly when compared with that of the HG group. Compared with that of the HG + BBR group, the apoptosis ratio and mitochondrial ROS level were significantly increased; the ATP levels were significantly reduced in the HG + BBR + miR-18a-5p mimics group.

### BBR Could Reduce STZ-Induced Renal Injury in DN Rats

**Figure 8:**
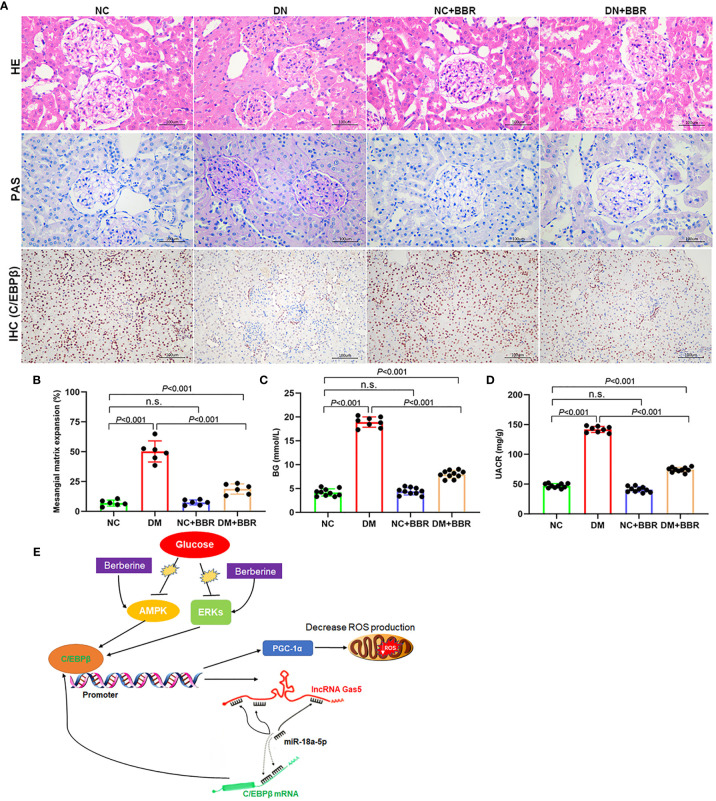
Effect of BBR on STZ induced renal injury in DN rats. **(A)** HE, PAS, and immunohistochemical staining results. **(B)** Quantification of mesangial matrix expansion determined as the percentage of PAS-positive area/glomerular area. **(C)** BG levels in different groups. **(D)** UACR levels in different groups. **(E)** Schematic diagram of positive feedback loop of BBR activated C/EBPβ/Gas5/miR-18a-5p. n.s., no significance.

We established DN rats model by STZ intraperitoneal injection. BBR was given intragastric administration for 8 weeks. HE and PAS staining results showed that the ECM increased, renal balloon dilated, and GBM thickened in the DN group; BBR significantly reduced glomerular matrix expansion (**Figures 8A, B**). Immunohistochemical results showed that the expression of C/EBPβ decreased in the renal tissue of DN rats; BBR could upregulate C/EBPβ in DN rats (**Figure 8A**). Blood glucose (BG) (**Figure 8C**) and UACR (**Figure 8D**) in DN rats were significantly increased (p < 0.001); BBR could downregulate BG and UACR. These suggested that BBR could upregulate C/EBPβ in kidney tissue of DN rats induced by STZ and improve the STZ-induced renal injury in DN rats.

## Discussion

The incidence rate of diabetes has gradually increased in recent years; diabetes patients worldwide are expected to rise to 55% of adults in the world by 2035. Diabetic complications involve heart, brain, kidney, and other important organs, with a high rate of death and disability; about 40% of patients have DN (26). DN, as the most typical microvascular complication of DM, develops progressively and irreversibly once it reaches the stage of dominant proteinuria and eventually becomes end-stage renal disease (ESRD). DN is characterized by progressive renal failure. The glomerular mesangial cells (GMCs) proliferation and the extracellular matrix (ECM) protein deposition in the glomerulus increase, which leads to the mesangial cells proliferation, ECM accumulation, the hypertrophy of mesangial cells, and finally the formation of glomerulosclerosis and atrophy (27). Long-term hyperglycemia can activate aldose reductase (AR) in the pathway of polyol metabolism in renal tissue and cause the activation of polyol signaling pathway. Activation of AR pathway leads to imbalance of oxidation and antioxidation of glomerular endothelial cells, resulting in neutrophils inflammatory infiltration, protease secretion increase, production of many oxidation intermediates, promotion of mitochondrial metabolism abnormality, and OS (28). On the one hand, OS can increase the secretion of angiotensin II, the pressure of glomerulus, the glomerular filtration rate, and formation of proteinuria and the thickening of GBM, and accelerate the progress of diabetic kidney disease (DKD) (29). On the other hand, OS can activate intracellular signaling pathways, such as c-JUN N-terminal kinase (JNK) and protein kinase C (PKC) pathways, and activate transcription factors nuclear factor kappa B (NF-κB) and activator protein 1 (AP-1), which accelerate ECM deposition and reduce extracellular matrix degradation, leading to glomerulosclerosis and renal fibrosis (30). OS can also cause insulin receptor damage, interfere with the insulin signal pathway of insulin binding with insulin receptor, and cause insulin resistance (31). Insulin resistance can increase the pressure gradient of glomerular capillaries, reduce the contraction of outflow arterioles, reduce hydrostatic pressure and permeability, and lead to glomerular hypertrophy and sclerosis (32).

BBR is extracted from *C. chinensis*; it is an isoquinoline alkaloid monomer. It can regulate many diseases such as hyperlipidemia, hypertension, inflammation, diarrhea, and tumor. Berberine has great therapeutic potential in diabetes and its complications, such as DN (5, 33). BBR can reduce the absorption of glucose in the intestine and produce hypoglycemic effect by increasing the utilization of glucose by fat cells, liver cells, and skeletal muscle cells (34–36). BBR can inhibit OS by increasing the superoxide dismutase mRNA expression (37). Hsu considered that BBR inhibited the action of OS by inhibiting Nrf-2 pathway and the activities of AMP-activated protein kinase (AMPK), PI3K/Akt, and p38 pathway, and activated the expression of antioxidant enzymes such as superoxide dismutase and glutathione, which delayed the formation of glomerulosclerosis and finally the process of renal fibrosis (38). lncRNAs play an important role in DN. However, it is not clear whether BBR plays a protective role in DN by regulating lncRNA expression. In this study, we observed the expression levels of 10 lncRNAs including Blnc1, PRINS, LINK-A, Tug1, Gas5, MALAT1, H19, ARAP1-AS2, ARAP1-AS1, and PVT1 (13–20) that have been reported to be related to the pathogenesis of DN after HG and BBR treatment. The results showed that HG could downregulate the expression of Gas5 in HK-2 cells, while BBR could counteract the inhibitory effect of HG on Gas5 expression. Gas5, located on chromosome 1q25, is a 5′-terminal oligopyrimidine class of genes. It can be transcribed into several small nucleolar RNAs (snoRNAs) and four splice variants of Gas5 mRNA. However, they cannot be translated into proteins due to the existence of stop codons. A current study showed that Gas5 could affect cell survival and proliferation (39). It was associated with a variety of tumors, such as colon cancer, melanoma, prostate cancer, bladder cancer, and Parkinson’s disease (40, 41). Gas5 in peripheral blood is associated with diabetes incidence rate. The decrease in expression level of Gas5 is related to insulin resistance; it is a high risk factor for diabetes (17). Gas5 could regulate the proliferation and fibrosis of mesangial cells by regulating miR-221/SIRT1 signaling pathway (42). It is not clear whether BBR plays a protective role in DN through regulating lncRNA expression. This study showed that the mitochondrial ROS levels increased significantly in HK-2 cells in HG environment; the cell apoptosis level in early stage was significantly increased. There was a feedback loop of C/EBPβ/Gas5/miR-18a-5p in HK-2 cells. HG could inhibit the C/EBPβ expression, downregulate the Gas5 expression, increase the miR-18a-5p expression level, further inhibit the C/EBPβ expression, lead to the inhibition of PGC-1α signal pathway, and cause the increase in mitochondrial ROS generation. BBR could promote the C/EBPβ expression in HK-2 cells treated by HG, upregulate the Gas5 expression, inhibit the miR-18a-5p expression, promote the activation of PGC-1α signal pathway, and inhibit the mitochondrial ROS level and the early apoptosis.

In recent years, many studies have found that in the progression of DN, there are many kinds of mitochondrial damage in the intrinsic cells of kidney, such as mitochondrial energy metabolism disorder, which suggests that mitochondrial energy metabolism plays an important role in the pathogenesis and progression of DN (43, 44). Disorder of mitochondrial energy metabolism can generate excessive ROS, and excessive ROS can promote the release of Cyt C from mitochondrial membrane space, activate caspase-9 and caspase-3 signal pathways, and cause apoptosis (45). PGC-1α is considered to be a central regulator of mitochondrial biosynthesis, which is widely expressed in the heart, brain, kidney, and other organs; it can increase the content of mitochondria and reduce ROS accumulation (24). Previous study have shown that the PGC-1α expression is regulated by a cAMP-regulated transcription factor C/EBPβ; C/EBPβ can bind CREs located between 2,756 and 2,752 bp of PGC-1α promoter and promote its expression (25). The decrease in PGC-1α expression in diabetic muscle may be the reason for the decrease in NRF-dependent metabolism and mitochondrial gene expression (25). In DN renal tissue, the levels of PGC-1α and AMPK decreased, accompanied by the decrease in mitochondrial content and complex enzyme activity (46). In addition, PKA plays an important regulatory role in the PGC-1α expression. ERK1/2 can phosphorylate and activate C/EBPβ, which is the key to cAMP-dependent C/EBPβ activation (47, 48). The activation of AMPK and ERK1/2 by BBR in diabetes has been confirmed in many studies (49–51); we found that BBR could activate the C/EBPβ expression in this study. PGC-1α is also a major transcriptional regulator of ROS scavenging enzymes, such as uncoupling protein 2, catalase, GSH-Px, and Mn-SOD2. The decreased expression of PGC-1α may promote oxidative stress (52–54). These results suggested that PGC-1α signaling pathway played a key role in the mitochondrial energy metabolism. We found that the expression level of PGC-1α decreased in HK-2 cells treated with HG, and the mitochondrial energy metabolism was disordered. While BBR could activate C/EBPβ to upregulate the Gas5 expression, Gas5 could further promote the C/EBPβ expression by inhibiting the miR-18a-5p expression through the ceRNA effect. Meanwhile, C/EBPβ could further activate the PGC-1α expression and correct the disorder of mitochondrial energy metabolism (**Figure 8**).

## Conclusions

In summary, BBR could alleviate the disorder of energy metabolism in HK-2 cells caused by HG. BBR regulated the energy metabolism of cell mitochondria, inhibited the generation of ROS, and reduced cell damage by activating the C/EBPβ/Gas5/miR-18a-5p positive feedback loop and the C/EBPβ/PGC-1α signal pathway. These events indicated that lncRNA Gas5 is directly involved in the molecular pathway of DN occurrence and BBR treatment, which may be used as a molecular marker for disease diagnosis and efficacy judgment.

## Data Availability Statement

The raw data supporting the conclusions of this article will be made available by the authors, without undue reservation.

## Ethics Statement

The studies involving human participants were reviewed and approved by The First Affiliated Hospital of USTC. The patients/participants provided their written informed consent to participate in this study. The animal study was reviewed and approved by The First Affiliated Hospital of USTC.

## Author Contributions

Conception and design: JZ and SY. Administrative support: JZ and SY. Provision of study materials or patients: JX, LL, LG, and YH. Collection and assembly of data: JX, LL, PX, and YX. Data analysis and interpretation: JX, PX, and SY. Manuscript writing: all authors. All authors contributed to the article and approved the submitted version.

## Funding

This study was supported by the Local Science and Technology Development Project Guide by The Central Government of China (2017070802D147) and The Anhui Provincial Natural Science Foundation (1508085SMH227).

## Conflict of Interest

The authors declare that the research was conducted in the absence of any commercial or financial relationships that could be construed as a potential conflict of interest.

## Publisher’s Note

All claims expressed in this article are solely those of the authors and do not necessarily represent those of their affiliated organizations, or those of the publisher, the editors and the reviewers. Any product that may be evaluated in this article, or claim that may be made by its manufacturer, is not guaranteed or endorsed by the publisher.

## Abbreviations

BBR, berberine; DM, diabetes mellitus; DN, diabetic nephropathy; ECM, extracellular matrix; ESRD, end-stage renal disease; FISH, fluorescence *in situ* hybridization; GBM, glomerular basement membrane; HG, high glucose; lncRNA, long noncoding RNA; NAFLD, nonalcoholic fatty liver disease; OS, oxidative stress; RTC, renal tubular cells; STZ, streptozotocin; UACR, urinary albumin-to-creatinine ratio.
